# Multifactorial Mechanisms of Tolerance to Ketoconazole in Candida albicans

**DOI:** 10.1128/spectrum.00321-21

**Published:** 2021-06-23

**Authors:** Yi Xu, Hui Lu, Shuo Zhu, Wan-Qian Li, Yuan-ying Jiang, Judith Berman, Feng Yang

**Affiliations:** a Department of Pharmacy, The 960 Hospital of PLA, Jinan, China; b Department of Pharmacology, Shanghai Tenth People’s Hospital, Tongji University School of Medicine, Shanghai, China; c Shmunis School of Biomedical and Cancer Research, The George S. Wise Faculty of Life Sciences, Tel Aviv University, Tel Aviv, Israel; d Department of Vascular Disease, Shanghai TCM-Integrated Hospital, Shanghai University of Traditional Chinese Medicine, Shanghai, China; Broad Institute

**Keywords:** antifungal tolerance, *CDR1*, calcineurin, *Candida albicans*, Hsp90, ketoconazole, V-ATPase, *VMA11*, antifungal resistance

## Abstract

Candida albicans is a prevalent opportunistic human fungal pathogen for which treatment is limited to only four main classes of antifungal drugs, with the azole and echinocandin classes being used most frequently. Drug tolerance, the ability of some cells to grow slowly in supra-MIC drug concentrations, decreases the number of available treatment options. Here, we investigated factors affecting tolerance and resistance to ketoconazole in C. albicans. We found both temperature and the composition of growth medium significantly affected tolerance with little effect on resistance. In deletion analysis of known efflux pump genes, *CDR1* was partially required for azole tolerance, while *CDR2* and *MDR1* were dispensable. Tolerance also required Hsp90 and calcineurin components; *CRZ1*, which encodes a transcription factor downstream of calcineurin, was required only partially. Deletion of *VMA11*, which encodes a vacuolar ATPase subunit, and concanamycin A, a V-ATPase inhibitor, abolished tolerance, indicating the importance of vacuolar energy transactions in tolerance. Thus, tolerance to ketoconazole is regulated by multiple factors, including physiological and genetic mechanisms.

**IMPORTANCE** Due to the ever-expanding range of invasive medical procedures and treatments, invasive fungal infections now pose a serious global threat to many people living in an immunocompromised status. Like humans, fungi are eukaryotic, which significantly limits the number of unique antifungal targets; the current arsenal of antifungal agents is limited to just three frontline drug classes. Additional treatment complexities result from the development of drug tolerance and resistance, which further narrows therapeutic options; however, the difference between tolerance and resistance remains largely unknown. This study demonstrates that tolerance and resistance are regulated by multiple genetic and physiological factors. It is prudent to note that some factors affect tolerance only, while other factors affect both tolerance and resistance. The complex underlying mechanisms of these drug responses are highlighted by the fact that there are both shared and distinct mechanisms that regulate tolerance and resistance.

## INTRODUCTION

Candida albicans is a common human commensal of the skin and gastrointestinal and genitourinary tracts; it is also the most prevalent human fungal pathogen, causing a range of infections from superficial infections of the skin to life-threatening systemic infections (1). There are four major chemical classes of antifungal drugs: azoles, echinocandins, polyenes, and flucytosine (2). Azoles are the most widely used, due to their broad spectrum of activity, favorable safety, and bioavailability. However, the fungistatic nature of azole antifungals promotes the rapid appearance of cells that acquire an increased ability to grow in the presence of inhibitory concentrations of the drug (3, 4), which can be due to antifungal resistance or tolerance. Polyenes, which are nephrotoxic, and the echinocandins, which recently transitioned to be the first-line drug of choice in western hospitals, are fungicidal to C. albicans but must be administered intravenously (5–7). Flucytosine is used only in drug combinations, as resistance often appears rapidly with monotherapy (8). When combined with fluconazole or amphotericin B, flucytosine provides some improvement in patient outcomes (9). Nevertheless, clinical options for antifungal drug therapy remain limited.

The incidence of clinical failures with azoles is increasing (10), yet the incidence of C. albicans isolates that are bona fide resistant has remained relatively constant (11). Some of this discrepancy may be due to the phenomenon of antifungal tolerance (12).

Antifungal resistance, measured using the MIC (minimum inhibitory drug concentration), is the ability to grow at drug concentrations that inhibit susceptible isolates. Antifungal tolerance is defined as the ability of drug-susceptible strains (usually a subpopulation of cells) to grow slowly at inhibitory drug concentrations (reviewed in reference 13) and can be measured using disk diffusion assays (DDAs) (12, 14) or broth microdilution assays (15). In DDAs, photographs of the plates are analyzed using the *diskImageR* pipeline. The degree of drug resistance is determined by the radius of inhibition (RAD), and tolerance is determined by the fraction of growth (FoG) within the zone of inhibition. Usually, 20% drug inhibition (RAD_20_ and FoG_20_) is used to measure resistance and tolerance, respectively (12, 14). Tolerance on plate-based assays is likely analogous to trailing growth in microdilution assays (reviewed in reference 13). Furthermore, high levels of fluconazole tolerance may play an important role in the failure to clear C. albicans and C. tropicalis infections (12, 16, 17).

Inhibitors of some cellular stress pathways are synergistic with fluconazole, primarily via their effect on tolerance rather than on resistance (12), suggesting that tolerance and resistance are regulated by distinct mechanisms. However, the literature generally has not distinguished between resistance and tolerance; thus, mechanisms that specifically affect tolerance have not been studied extensively.

Ketoconazole (KCZ) is an imidazole. Like other azoles, KCZ inhibits the cytochrome P450 14α-demethylase, a key enzyme in the ergosterol biosynthetic pathway in fungi (18). In addition, KCZ alone or in combination with other agents exhibits promising anticancer efficacy against multiple cancers (19).

In this study, we investigated factors that regulated tolerance to KCZ in C. albicans. We tested the effect of temperature and medium, the role of genes encoding drug efflux pumps, and the role of Hsp90, calcineurin, and V-ATPase on tolerance. We found tolerance of KCZ was regulated by multiple factors.

## RESULTS

### Temperature and medium regulate tolerance to KCZ in C. albicans.

**FIG 1:**
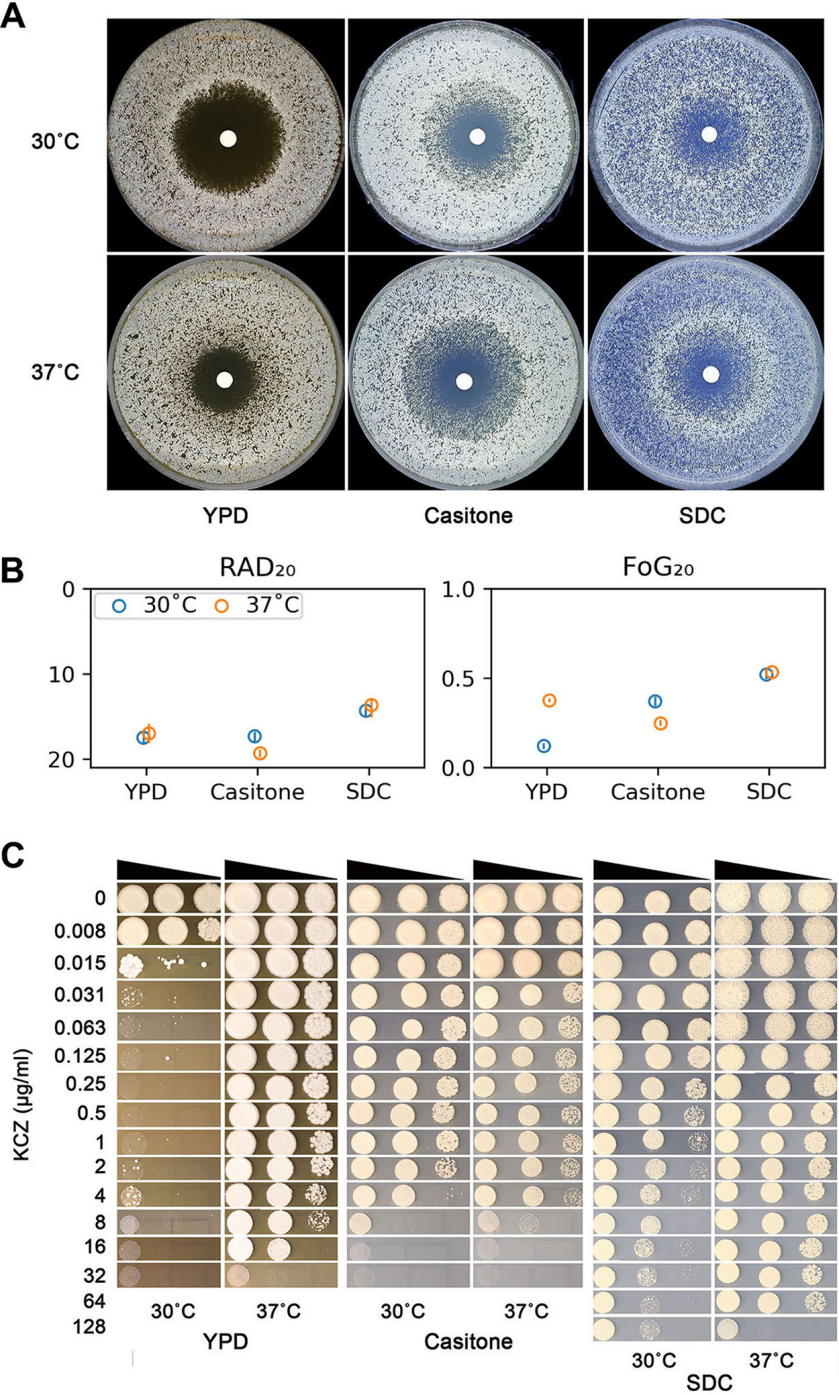
Temperature and medium effects on ketoconazole tolerance. (A) The laboratory strain SC5314 was tested at 30°C and 37°C on YPD, Casitone, and SDC medium with disk diffusion assays. Each disk contained 50 μg of ketoconazole. Pictures of the plates were analyzed using *diskImageR*. RAD_20_ was determined at 24 h, and FoG_20_ was determined at 48 h. The RAD_20_ and FoG_20_ values were presented as point plots using a custom Python script. (B) The circles represent the means, and the vertical lines inside the circles represent the standard deviations from three biological repeats. Spot assays were also performed at 30°C and 37°C on the same medium supplemented with ketoconazole. (C) The plates were incubated for 48 h and then photographed.

The reference strain SC5314 was tested with disk diffusion assays (DDA) and spot assays on YPD, Casitone, and SDC plates at 30°C and 37°C. On YPD plates, the RAD_20_ values of SC5314 at 30°C and 37°C were 17.50 ± 0.71 and 17.00 ± 1.41, respectively. On Casitone plates, the RAD_20_ was 17.33 ± 0.58 and 19.33 ± 0.58 at 30°C and 37°C, respectively. On SDC plates, the RAD_20_ was 14.33 ± 0.58 and 13.67 ± 1.15 at 30°C and 37°C, respectively (Fig. 1A and B). Thus, temperature does not have obvious effect on resistance on YPD, Casitone, or SDC plates.

On YPD plates, the zone of inhibition was clear at 30°C, but at 37°C there was obvious growth of colonies in the outer edge of the inhibition zone (Fig. 1A). Consistent with this, *diskImageR* analysis indicated the FoG_20_ values at 30°C and 37°C were 0.12 ± 0.01 and 0.38 ± 0.01, respectively (Fig. 1B). However, on both Casitone and SDC plates, at both 30°C and 37°C, there were obvious colonies growing in the zone of inhibition (Fig. 1A). The Fog_20_ values on Casitone plates at 30°C and 37°C were 0.37 ± 0.02 and 0.25 ± 0.01, respectively, and on SDC plates the values were 0.52 ± 0.02 and 0.53 ± 0.01, respectively (Fig. 1B). Thus, on YPD, SC5314 was tolerant at 37°C but not at 30°C. On Casitone and SDC, SC5314 was tolerant at both 30°C and 37°C.

Next, we examined the extent that tolerance facilitated growth in the presence of KCZ. Spot assays were used to measured growth over a range of KCZ concentrations (0.008 μg to 128 μg/ml) on different media and temperatures. Although the RAD_20_ values did not change obviously at different temperatures on YPD, the ability to grow in the presence of KCZ differed obviously (Fig. 1C). At 30°C, the growth of SC5314 was obviously inhibited at 0.015 μg/ml KCZ, while at 37°C, only 32 μg/ml KCZ obviously inhibited growth. Since SC5314 was not tolerant at 30°C but was tolerant at 37°C, we conclude tolerance enables growth at supra-MICs of KCZ.

Similarly, on Casitone and SDC plates, SC5314 was tolerant at both 30°C and 37°C. Spot assay indicated that, on Casitone, at both 30°C and 37°C, the growth was obviously inhibited at 8 μg/ml KCZ. On SDC, SC5314 could grow at 128 μg/ml KCZ at 30°C and was obviously inhibited only at 128 μg/ml KCZ at 37°C (Fig. 1C). Thus, tolerance determines the ability of growth in the presence of KCZ.

### *CDR1* is partially required for KCZ tolerance.

To investigate the role of the genes encoding efflux pumps in tolerance, we constructed homozygous deletions of *CDR1*, *CDR2*, and *MDR1* and tested these deletion strains by DDA and spot assay on YPD medium at 30°C and 37°C.

**FIG 2:**
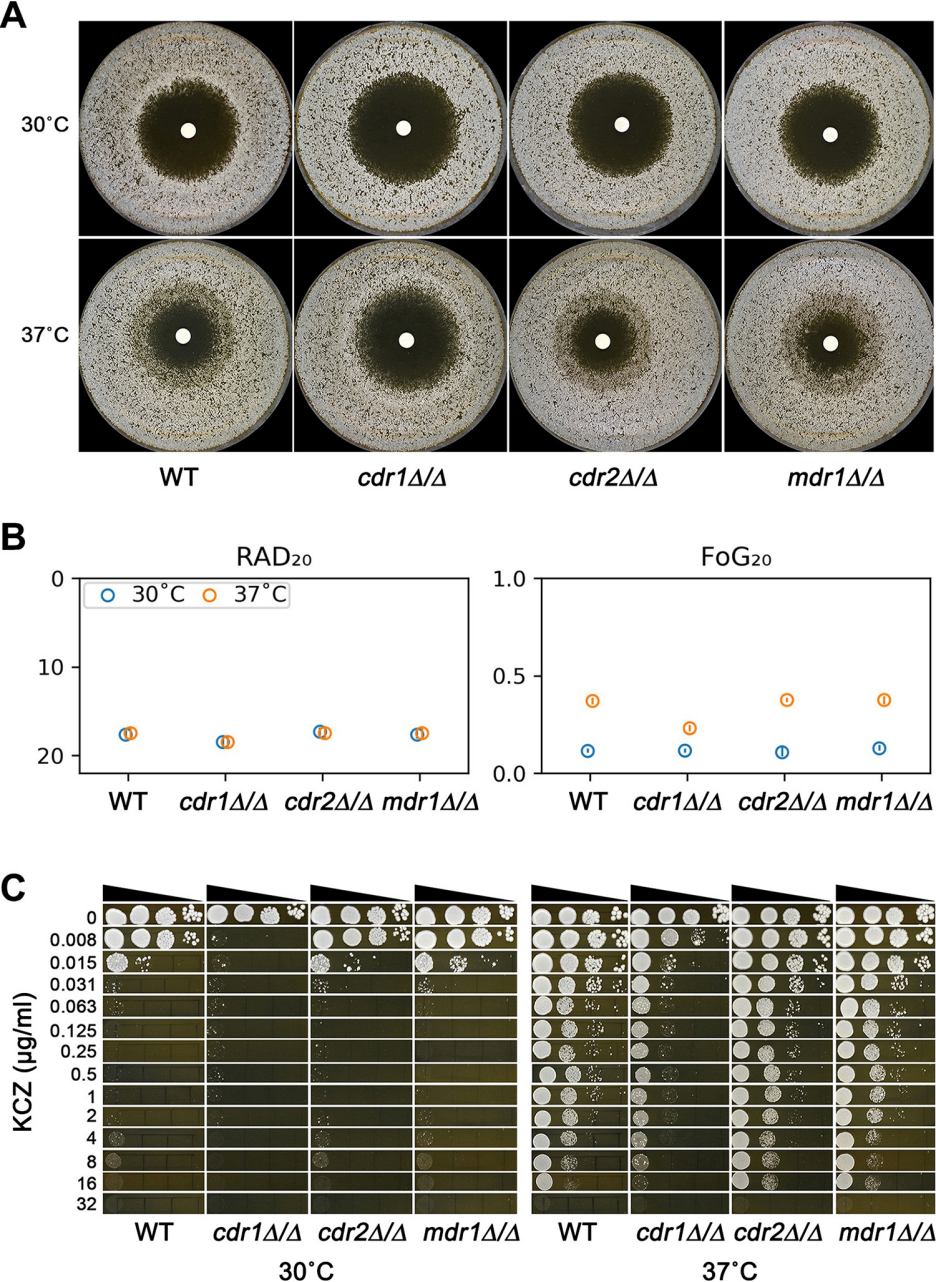
Role of *CDR1*, *CDR2*, and *MDR1* in ketoconazole tolerance. (A) SC5314-derived *cdr1*Δ/Δ, *cdr2*Δ/Δ, and *mdr1*Δ/Δ strains were tested with disk diffusion assay at 30°C and 37°C on YPD medium. Each disk contained 50 μg of ketoconazole. The pictures of the plates were analyzed using *diskImageR*. (B) The circles represent the means, and the vertical lines inside the circles represent standard deviations from three biological repeats. (C) Spot assays were performed on YPD medium supplemented with ketoconazole.

At 30°C, strains with homozygous deletions of *CDR1*, but not *CDR2* or *MDR1*, had slightly increased RAD_20_ values. The RAD_20_ values of the parent, *cdr1*Δ/Δ, *cdr2*Δ/Δ, and *mdr1*Δ/Δ strains were 17.67 ± 0.58, 18.50 ± 0.71, 17.33 ± 0.58, and 17.67 ± 0.58, respectively (Fig. 2A and B). Since none of these strains were tolerant at 30°C, as indicated by clear zones of inhibition (Fig. 2A) and low FoG_20_ values (Fig. 2B), the ability to grow in the presence of KCZ was determined by RAD_20_. Spot assay indicated that growth of the wild-type (WT), *cdr2*Δ/Δ, and *mdr1*Δ/Δ strains was inhibited by 0.015 μg/ml KCZ, and the *cdr1*Δ/Δ strain was inhibited by 0.008 μg/ml KCZ (Fig. 2C).

At 37°C, the RAD_20_ values of the parent and the deletion strains were similar to the values at 30°C (Fig. 2B). The FoG_20_ values of the parent, *cdr1*Δ/Δ, *cdr2*Δ/Δ, and *mdr1*Δ/Δ strains were 0.37 ± 0.01, 0.23 ± 0.01, 0.38 ± 0.01, and 0.38 ± 0.02, respectively (Fig. 2B). Thus, deletion of *CDR1*, but not *CDR2* or *MDR1*, partially decreased tolerance to KCZ at 37°C on YPD. Consistent with this, spot assay indicated growth of parent, *cdr2*Δ/Δ, and *mdr1*Δ/Δ strains was inhibited at 32 μg/ml KCZ, while growth of the *cdr1*Δ/Δ strain was inhibited at 0.015 μg/ml KCZ, and it could still grow, although to a lesser degree, at 0.015 μg/ml to 8 μg/ml KCZ (Fig. 2C). Taken together, deletion of *CDR1* partially decreases tolerance to KCZ at 37°C. Deletion of *CDR2* or *MDR1* does not alter tolerance to KCZ at 37°C.

### Hsp90 and calcineurin are required for KCZ tolerance.

**FIG 3:**
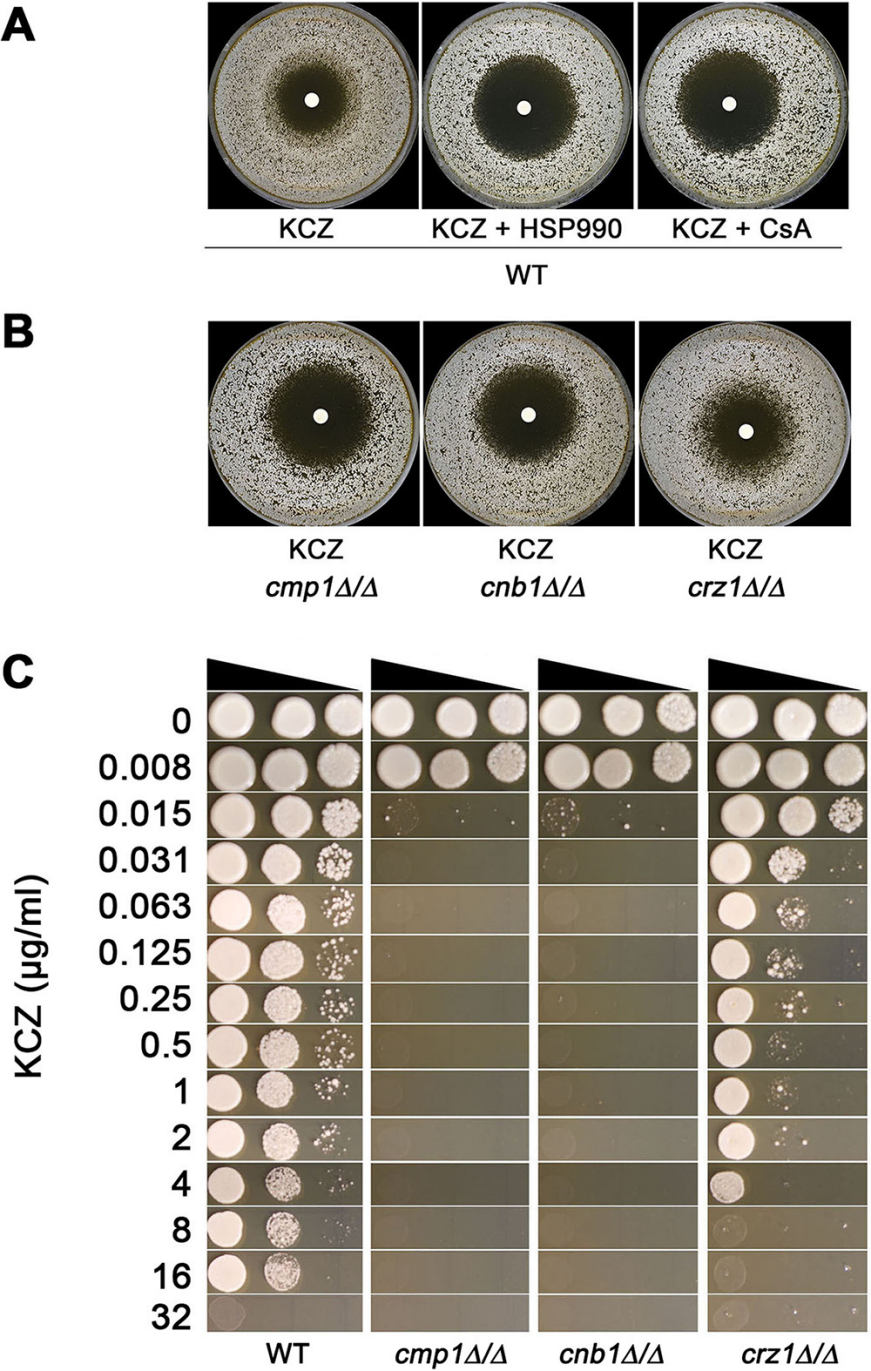
Role of Hsp90 and calcineurin in ketoconazole tolerance. (A) SC5314 was tested with disk diffusion assay at 37°C on YPD or YPD supplemented with Hsp90 inhibitor NVP-HSP990 (HSP990; 2 μg/ml) or calcineurin inhibitor cyclosporine (CsA; 1 μg/ml). (B) SC5314-derived *cmp1*Δ/Δ, *cnb1*Δ/Δ, and *crz1*Δ/Δ strains were tested with disk diffusion assays on YPD medium. In panels A and B, each disk contained 50 μg of ketoconazole. (C) Spot assay was performed at 37°C to compare the wild-type strain SC5314 and homozygous deletion strains of genes *CMP1*, *CNB1*, and *CRZ1* using YPD medium supplemented with ketoconazole.

Previously, it was demonstrated that Hsp90 and calcineurin are required for tolerance to fluconazole (12). We asked if they were also required for KCZ tolerance. Growing on YPD at 37°C, the tolerance to KCZ in SC5314 was abolished by Hsp90 inhibitor NVP-HSP990 (HSP990) and calcineurin inhibitor cyclosporine (CsA), as indicated by clear zones of inhibition (Fig. 3A). Calcineurin has two subunits, one catalytic subunit and one regulatory unit, which are encoded by *CMP1* and *CNB1*, respectively. The *CRZ1* gene encodes a downstream transcription factor (20). We found homozygous deletions of *CMP1* and *CNB1* totally abolished the tolerance, but homozygous deletion of *CRZ1* only partially abolished tolerance. (Fig. 3B). Spot assay indicated the growth of *cmp1*Δ/Δ and *cnb1*Δ/Δ strains was inhibited at 0.015 μg/ml KCZ. Although the growth of the *crz1*Δ/Δ strain was obviously inhibited at 0.063 μg/ml KCZ, it could still grow at 0.063 μg/ml to 4 μg/ml, while the wild-type strain could grow at 16 μg/ml KCZ (Fig. 3C). Thus, *CMP1* and *CNB1* are required for KCZ tolerance, and *CRZ1* is partially required.

### V-ATPase is required for KCZ tolerance.

**FIG 4:**
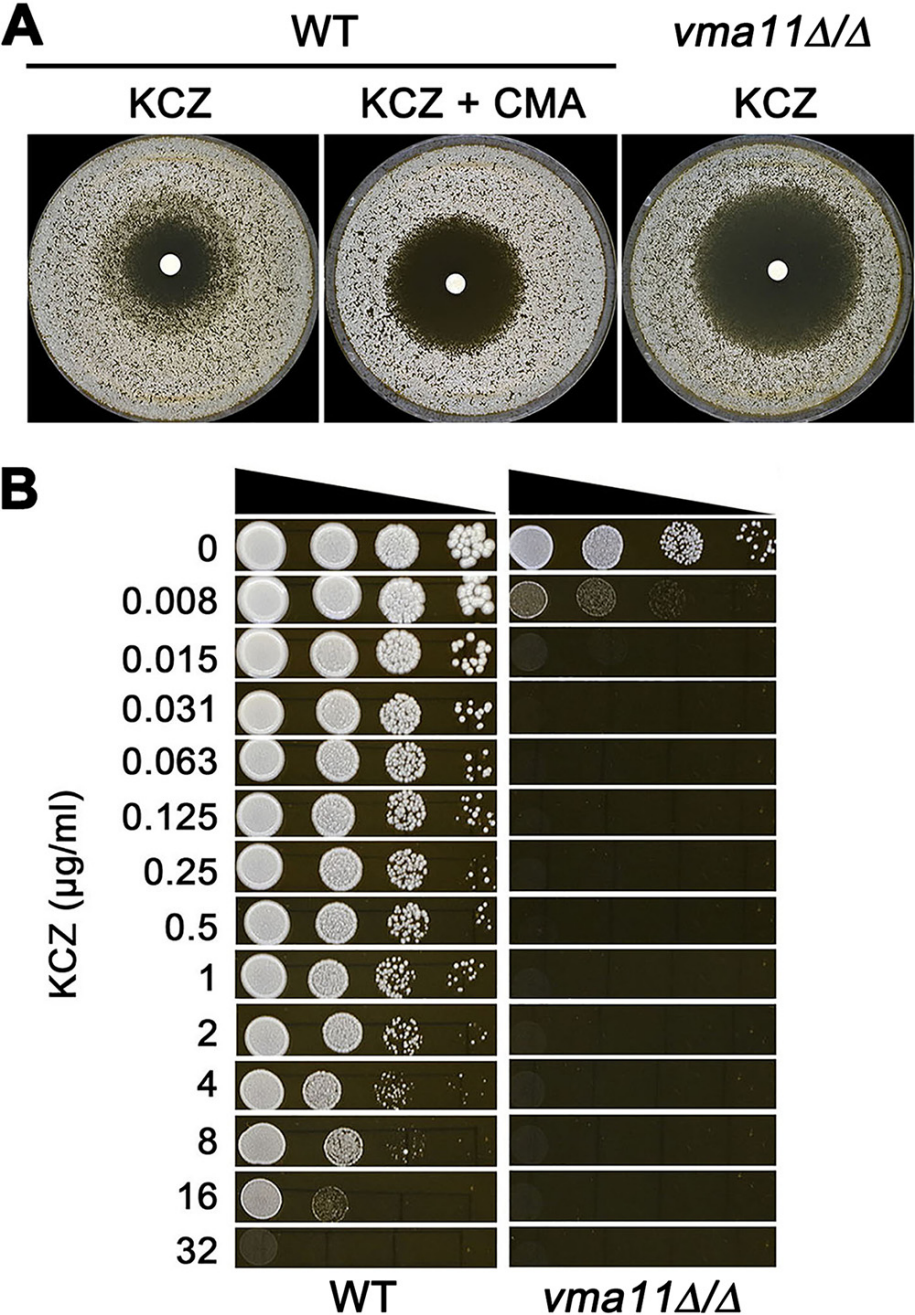
Role of V-ATPase in ketoconazole tolerance. (A) SC5314 was tested with disk diffusion assay on YPD plate or YPD plate supplemented with V-ATPase inhibitor concanamycin A (CMA; 0.2 ng/ml). The SC55314-derived *vma11*Δ/Δ strain was also tested on YPD. Each disk contained 50 μg of ketoconazole. (B) *vma11*Δ/Δ strain was compared to the wild-type strain SC5314 for the ability to grow in the presence of ketoconazole on YPD medium. In panels A and B, the plates were incubated at 37°C for 48 h.

To investigate the role of V-ATPase in KCZ tolerance, we first tested the effect of the V-ATPase inhibitor concanamycin A (CMA). Tested on YPD at 37°C, SC5314 grown on a YPD plate supplemented with 0.2 μg/ml CMA lost tolerance to KCZ (Fig. 4A). Next, we deleted *VMA11*, which encodes the c' subunit of the V0 subcomplex of the V-ATPase. We found the homozygous deletion strain had an obviously big and clean zone of inhibition, with a RAD_20_ value of 22.00 ± 1.41 and FoG_20_ value of 0.09 ± 0.01 (Fig. 4A), indicating deletion of *VMA11* caused both loss of tolerance and a decrease in resistance to ketoconazole. Spot assay on YPD plates at 37°C also indicated that while the growth of the parent was inhibited at 32 μg/ml KCZ, growth of the *vma11*Δ/Δ strain was inhibited at 0.008 μg/ml KCZ (Fig. 4B). Thus, compromising the function of the V-ATPase through pharmacological inhibition or gene deletion abolishes KCZ tolerance.

## DISCUSSION

Previously, it was demonstrated that multiple factors regulated tolerance but not resistance to fluconazole in C. albicans (12, 14). Both physiological (e.g., pH) and genetic (e.g., overexpression of *CRZ1* and *GZF3*) factors have been identified as mediators of fluconazole tolerance. In addition, deletion of *crz1* has been shown to reduce fluconazole tolerance without changing the MIC in several C. albicans genetic backgrounds (21). In this study, we tested more factors that regulated tolerance to KCZ, an imidazole antifungal drug, in C. albicans. Tolerance was measured by DDAs and spot assays. The laboratory strain SC5314 was tested using different media at different temperatures. In order to test the role of some genes in tolerance, we used both pharmacological inhibitors of the gene product and homozygous deletion of the genes.

Temperature is an important environmental factor that modulates C. albicans physiological characteristics (22), morphogenesis (23), phenotypic switching (24), virulence (25), and resistance to antifungal drugs (26). Compared to growth at lower temperatures (25°C and 30°C), C. albicans grown on YPD at high temperature (37°C and 42°C) was more tolerant to the cell wall stressor calcofluor white (27). In another study, Saccharomyces cerevisiae grown on synthetic defined (SD) medium and C. albicans grown in synthetic RPMI medium were more tolerant to fluconazole at 30°C than at 39°C (26).

In addition to temperature, growth medium also has an influence on drug resistance (28–30), planktonic growth, adhesion, and biofilm formation (31) in bacteria. In C. albicans, medium modulates antifungal drug resistance, biofilm formation, and virulence (32, 33). In Aspergillus fumigatus, medium influences glucan synthesis and, thus, the efficacy of echinocandins, which are inhibitors of β-1,3-glucan synthase (34).

In this study, we found, on nutrient-rich medium YPD, SC5314 tolerated the drug only at 37°C. However, on less rich media, Casitone and SDC, SC5314 was tolerant at both 30°C and 37°C. We speculate tolerance is enabled by stress responses, including thermal and nutrient stresses.

Increased drug efflux is a key mechanism of drug resistance in bacteria (35), fungi (36), and tumor cells (37). The extent of fluconazole tolerance is inversely correlated with intracellular levels of fluconazole in C. albicans (12). In the C. albicans genome, *CDR1* and *CDR2* encode the multidrug transporter of the ABC superfamily. *MDR1* encodes the multidrug resistance protein of the major facilitator superfamily (38). In this study, we found, in the laboratory strain SC5314, homozygous deletions of *CDR2* or *MDR1* did not have an obvious influence on resistance or tolerance to KCZ. At 30°C, SC5314 was not tolerant on YPD, and homozygous deletion of *CDR1* slightly increased susceptibility to KCZ, as indicated by an elevated RAD_20_ value. At 37°C, SC5314 was tolerant on YPD, and homozygous deletion of *CDR1* only slightly decreased tolerance, as indicated by decreased FoG_20_ value, and less growth on drug plates, as shown by the spot assay. Thus, *CDR1* is partially required for tolerance.

The molecular chaperone Hsp90 functions in concert with cochaperones to regulate stability and activation of client proteins, many of which are signal transducers (39). Hsp90 enables azole and echinocandin resistance via calcineurin, and the downstream effector Crz1 plays a partial role (40, 41). Combining either geldanamycin (Hsp90 inhibitor) or cyclosporine (calcineurin inhibitor) with the fungistatic antifungal fluconazole renders a fungicidal response that abolished tolerance but had little effect on resistance. Overall, this indicates that Hsp90 and calcineurin are required for tolerance to fluconazole (12, 42). Calcineurin also controls some phenotypes independently on Crz1. For example, in C. albicans, the Rim101/PacC pH‐sensing pathway acts in parallel to Crz1, via calcineurin, to adapt to alkaline pH (43). In Cryptococcus neoformans, in response to thermal stress, although Crz1 acts downstream of calcineurin to govern gene expression, calcineurin also controls the expression of some genes independently of Crz1 (44). In S. cerevisiae, calcineurin causes depolarization of the actin cytoskeleton independently of Crz1 (45). Hsp90 and calcineurin are also required for fluconazole tolerance (12). Similarly, we found inhibitors of Hsp90 and calcineurin completely abolished KCZ tolerance but had little effect on resistance. *CMP1* and *CNB1*, which encode catalytic and regulatory subunits of calcineurin, respectively, were required for KCZ tolerance. Homozygous deletions of *CMP1* or *CNB1* completely abolished tolerance. However, *CRZ1* was partially required, indicating there are other pathways controlling KCZ tolerance in parallel to Crz1 via calcineurin. Thus, Hsp90 and calcineurin are required for KCZ tolerance, and Crz1 is partially required. Other calcineurin downstream effectors controlling KCZ tolerance remain to be identified.

Vacuolar-type ATPases (V-ATPase) are ubiquitous membrane-embedded ATP hydrolysis-driven proton pumps of all eukaryotic cells. V-ATPases are the primary driving force of the acidic pH of the vacuolar system and are essential for many fundamental cellular processes (46). V-ATPases are commonly activated in resistant and multidrug-resistant cancer cells (reviewed in reference 47). Inhibition of V-ATPase sensitizes tumor cells to anticancer drugs (48, 49). V-ATPases are also required for drug resistance in fungi, including S. cerevisiae, C. albicans, and C. glabrata (50, 51). V-ATPases are heteromultimeric enzymes consisting of a cytosolically oriented catalytic V_1_ domain and a membrane-bound proton-translocating V_o_ domain. Each complex is complexed with several subunits, and each subunit has multiple isoforms (46). Although the function of V-ATPase is conserved in all eukaryotic cells, the existence of the fungal-specific subunit C', encoded by *VMA11*, supports the potential of the V-ATPase as an antifungal drug target (52). In this study, the KCZ tolerance in the wild-type strain, which was enabled by high temperature, was abolished by the V-ATPase inhibitor concanamycin A. Furthermore, deletion of *VMA11* in the laboratory strain SC5314 also abolished tolerance and decreased resistance to KCZ, indicating the fungal-specific *VMA11* is a good target of both tolerance and resistance.

Taken together, KCZ tolerance enables growth irrespective of resistance level. KCZ tolerance is regulated by both physiological and genetic mechanisms. It will be interesting to investigate if tolerance to other antifungals, such as echinocandins, is regulated by similar or distinct mechanisms.

## MATERIALS AND METHODS

### Strains and growth conditions.

Strains used in this study are listed in Table S1 in the supplemental material. Strains were stored in 15% glycerol at −80°C. Media used in this study include yeast extract-peptone-dextrose (YPD) agar plates (1% [wt/vol] yeast extract, 2% [wt/vol] peptone, 2% [wt/vol] d-glucose, and 2% [wt/vol] agar), Casitone agar plates (0.9%[wt/vol] Casitone, 0.5% [wt/vol] yeast extract, 1.15% sodium citrate dihydrate [wt/vol], 2% [wt/vol] glucose, 2% [wt/vol] d-glucose, and 2% [wt/vol] agar), and SDC agar plates (0.67% [wt/vol] yeast nitrogen base without amino acids, 2% [wt/vol] d-glucose, 0.2% [wt/vol] complete amino acid mixture, and 2% [wt/vol] agar). For the selection of gene knockout strains and gene overexpression strains, YPD agar containing 400 μg/ml nourseothricin (NAT; Werner BioAgents) medium was used (YPD+NAT).

The same medium was used for growing cells and doing tests. The medium and temperature used in each experiment are specified in the figure legends.

Drugs were dissolved in dimethyl sulfoxide (DMSO) and stored at −20°C. Concentrations of the drugs used in each experiment are specified in the figure legends.

### Disk diffusion assay.

The CLSI M44-A2 guidelines (53) for antifungal disk diffusion susceptibility testing were followed, with slight modifications. Strains were grown on agar plates, cell density was adjusted to 1 × 10^6^ cells/ml as described above, and 100 μl of cell suspension was plated on plates. One paper disk (GE Healthcare, USA) supplemented with 50 μg ketoconazole was placed in the center of each plate. The plates were then incubated for 24 h and 48 h and photographed. Photographs were analyzed using the *diskImageR* pipeline (14). Means and standard deviations of RAD_20_ and FoG_20_ for three biological repeats were presented as point plots using a custom python script.

### Spot assay.

Strains were streaked onto plates and incubated for 24 h. Several colonies were randomly chosen and suspended in distilled water. Cell densities were determined by using a hemocytometer and adjusted to 1 × 10^7^ cells/ml. Serial 10-fold dilutions of cell suspension were spotted (3 μl/spot) on plates supplemented with the drugs. The plates were incubated for 48 h and then photographed.

### Gene deletions.

Gene deletions were performed as described previously (54). Primers used for deletions are listed in Table S2. The *NAT1* flipper gene deletion cassette was amplified from plasmid pJK863 (55). Approximately 500-bp upstream and 500-bp downstream regions of the gene to be deleted were amplified using the genomic DNA of SC5314 as the template. The upstream region of the gene was fused by PCR to the 5′ region of the cassette, and the downstream region of the gene was fused to the 3′ region of the cassette. The upstream and downstream fusion products for each gene were then simultaneously transformed in C. albicans by following the lithium acetate method (56). Transformants were selected on YPD plates supplemented with 400 μg/ml NAT. Diagnostic PCR using primers that annealed outside the flanking homologous regions of the gene was performed to confirm the replacement of the gene with the *NAT1* flipper cassette. The *NAT1* flipper was evicted by streaking the clones on yeast nitrogen base-bovine serum albumin plates.
