# Supplementary material for: Multifactorial Mechanisms of Tolerance to Ketoconazole in Candida albicans
Source: Microbiol Spectr. 2021 Jun 23;9(1):10.1128/spectrum.00321-21. doi: 10.1128/spectrum.00321-21 (PMC8552639; doi:10.1128/spectrum.00321-21)
Supplement: SUPPLEMENTAL FILE 1 — Supplemental material. Download SPECTRUM00321-21_Supp_1_seq1.pdf, PDF file, 0.1 MB [file spectrum00321-21_supp_1_seq1.pdf]

Table S1. Strains used in this study

Strain	Genotype	Parent	Source
SC5314	Wild type		(1)
YCA779	cdr1::FRT/cdr1::NAT1 flp	SC5314	This study
YCA776	cdr2::FRT/cdr2::NAT1 flp	SC5314	This study
YCA652	mdr1::FRT/mdr1::NAT1 flp	SC5314	This study
YCA641	cmp1::FRT/cmp1::NAT1 flp	SC5314	This study
YCA623	cnb1::FRT/cnb1::NAT1 flp	SC5314	This study
YCA736	crz1::FRT/crz1::NAT1 flp	SC5314	This study
YCA1072	vma11::FRT/vma11::NAT1 flp	SC5314	This study

References

1. Noble SM, Johnson AD. 2005. Strains and strategies for large-scale gene deletion studies of the diploid human fungal pathogen Candida albicans. Eukaryot Cell 4:298-309.

Table S2. Sequences of primers used in this study

Primer name	Primer sequence (5' to 3')
Gene deletions	
CaCDR1-US-F	GGAGCTCAACGGAAAATTG
NAT1-CaCDR1-US-R	GTATAGGAACTTCCTCGAGGGCTTCTATTGGTGTGGGATCC
NAT1-CaCDR1-DS-F	AGATCCACTAGTTCTAGAGCGGGTAGACGTGGTGGTTAGTGGTC
CaCDR1-DS-R	CAGATGAGAAACACTTTTTCCC
CaCDR2-US-F	CCCTGTTGAAATTTCCACTC
NAT1-CaCDR27-US-R	GTATAGGAACTTCCTCGAGGGCAATCATTGTGGTATACATCGG
NAT1-CaCDR2-DS-F	AGATCCACTAGTTCTAGAGCGGCATTGGTAGCACCTTTCACC
CaCDR2-DS-R	CTGCCATGGTAATGATGTTG
CaMDR1-US-F	GCTACCAATTAATCACAACGG
NAT1-CaMDR1-US-R	GTATAGGAACTTCCTCGAGGGGTTTGGTGTCTGATTCTTGC
NAT1-CaMDR1-DS-F	AGATCCACTAGTTCTAGAGCGGCGTGTTGCTTTTGCTTACG
CaMDR1-DS-R	GGCTAAGGTTATCCGTGTTC
CaCMP1-US-F	CGACACACATCAACAGAGTACAC
NAT1-CaCMP1-US-R	GTATAGGAACTTCCTCGAGGGTTGAGAAGGGGTAAAGGGG
NCAT1-CaCMP1-DS-F	AGATCCACTAGTTCTAGAGCGGGTAGAAAGTAGAGTGCGCGG
CaCMP1-DS-R	GCAAAGTCATCAAGTTGGG
CaCNB1-US-F	CTGGTGGCTCATTCTTTGTC
NAT1-CaCNB1-US-R	GTATAGGAACTTCCTCGAGGGGGGTTGAAAGGTACGATGG
NAT1-CaCNB1-DS-F	AGATCCACTAGTTCTAGAGCGGGGTCAATACCTGCTGTAACACC
CaCNB1-DS-R	CTTTCACATCAATTTTGGGAG
CaCRZ1-US-F	CAAGCTAATCAAGCTTACCAAG
NAT1-CaCRZ1-US-R	GTATAGGAACTTCCTCGAGGGGTTAGGGGAAATGAAATGACC
NAT1-CaCRZ1-DS-F	AGATCCACTAGTTCTAGAGCGGCGATAGTTATTCCTGTTGGTTG
CaCRZ1-DS-R	CTTCTTTTTCTGCGTGTGTG
CaVMA11-US-F	GAGAGAGAAAAAATGGTCGTG
NAT1-CaVMA11-US-R	GTATAGGAACTTCCTCGAGGGGATGAATACCGATTGTGTGG
NAT1-CaVMA11-DS-F	AGATCCACTAGTTCTAGAGCGGGTTTCCATTTTATCATCTTCTGG
CaVMA11-DS-R	CGGTATCACAACAAATTGCC
Diagnostic PCR for deletions	
CaCDR1-USD-F	GCACACACACACAAACACAC
CaCDR1-DSD-R	GACGTACATTGAAGGTTTGG
CaCDR2-USD-F	CACCTTTGTCTCCATATTCGTC

CaCDR2-DSD-R	CTTGGTGTTATTGGTGGTGG
CaMDR1-USD-F	CGTTTAGTTGTTCCCAATCG
CaMDR1-DSD-R	GACACAAAACACGTACTTCGC
CaCMP1-USD-F	GTTGGTTGGTTGACGAAATAG
CaCMP1-DSD-R	GGATCAAACTGAAGAACTCGTG
CaCNB1-USD-F	GGAAGAGAATTGAACAGGTTGT
CaCNB1-DSD-R	GTGCAGATCTATAGCCATTCC
CaCRZ1-USD-F	CCATCATATTTACCACAATTGG
CaCRZ1-DSD-R	GATGATGATGATGGAACCG
CaVMA11-USD-F	CTTGTGATGTGGCAAAGTTC
CaVMA11-DSD-R	GATGCATTGGCATTGTTG
